# Brassinosteroids as Central Regulators of Plant Growth, Stress Tolerance, and Agricultural Resilience

**DOI:** 10.3390/plants15172582

**Published:** 2026-08-25

**Authors:** Rahmatullah Jan, Shahzad Iqbal, Sajad Ali, Kyung-Min Kim

**Affiliations:** 1 Coastal Agriculture Research Institute, Kyungpook National University, Daegu 41566, Republic of Korea; rahmat2021@knu.ac.kr; 2 Department of Semiconductor Engineering, Gachon University, Seongnamdaero, Sujeong-Gu, Seongnam 13120, Republic of Korea; 3 Department of Biological Sciences, College of Science, King Faisal University, Al-Ahsa 31982, Saudi Arabia; 4 Department of Applied Biosciences, Graduate School, Kyungpook National University, Daegu 41566, Republic of Korea

**Keywords:** brassinosteroid, stress tolerance, abiotic stress, biotic stress, hormone cross talk, crop improvement, climate-resilient crop

## Abstract

Brassinosteroids (BRs) are essential steroidal phytohormones that regulate plant growth, development, and responses to environmental stresses. Recent studies have demonstrated the important roles of BRs in enhancing plant tolerance to abiotic stresses, including drought, salinity, temperature extremes, heavy metal toxicity, and oxidative stress, as well as biotic stresses caused by pathogens and herbivores. This review summarizes current advances in BR biosynthesis, metabolism, transport, and signaling pathways, focusing on key components that mediate stress adaptation. We discuss the physiological and molecular mechanisms through which BRs improve stress tolerance, including regulation of antioxidant defense, ion homeostasis, osmotic adjustment, and stress-responsive gene expression. Particular attention is given to the extensive cross talk between BRs and other phytohormones, such as abscisic acid, jasmonic acid, salicylic acid, ethylene, auxin, and gibberellins, which enables plants to balance growth and defense under adverse conditions. Furthermore, we highlighted the potential applications of BRs in crop improvement through exogenous treatments, genetic engineering, and genome-editing approaches. However, the effectiveness of BR-based strategies is highly dependent on crop species, developmental stage, stress type, BR concentration, application method, and environmental conditions. In addition, excessive BR accumulation or application may result in undesirable growth responses, and further multi-location field validation is required before widespread agricultural implementation. Finally, we discuss emerging research trends, current knowledge gaps, and future perspectives for exploring BR signaling to develop climate-resilient crops. Overall, BRs represent promising targets for improving crop stress resilience; however, optimizing BR-mediated strategies and validating their long-term performance under diverse field conditions will be essential for their successful application in sustainable agriculture.

## 1. Introduction

### 1.1. Global Impact of Biotic and Abiotic Stresses on Plant Growth and Yield

Agriculture productivity is increasingly threatened by a wide range of biotic and abiotic stresses that severely limit plant growth, development, and crop yield worldwide. Abiotic stresses such as drought, salinity, extreme temperatures, heavy metal toxicity, and nutrient deficiency are among the primary environmental constraints affecting crop performance, particularly under changing climate conditions. It is estimated that abiotic stresses alone can reduce global crop yields by more than 50%, posing a significant challenge to food security and sustainable agriculture [1]. Climate change has further intensified the frequency and severity of stress events, including prolonged drought, heat waves, and soil salinization, thereby exacerbating agricultural losses and threatening food production system [2]. In addition to abiotic factors, biotic stresses caused by pathogens, insects, and herbivores represent another major source of crop damage and economic loss. Plant diseases alone are responsible for approximately 20 to 40% reduction in global crop yield annually, highlighting the urgent need for effective strategies to enhance plant resistance and resilience [3,4]. Combined biotic and abiotic stresses often exert synergistic effects that intensify physiological damage and reduce crop productivity. Understanding the molecular mechanisms underlying these responses is therefore essential for developing climate resilient crops.

### 1.2. Overview of Plant Hormone-Mediated Stress Responses

Plants perceive environmental stimuli through complex signaling pathways in which phytohormones coordinate growth, development, and stress responses. Major hormones, including ABA, SA, JA, ethylene, gibberellins, cytokinins, and brassinosteroids, regulate these processes through interconnected signaling networks [5]. Their specific interactions and crosstalk are discussed in detail in Section 6.

### 1.3. Discovery and Classification of Brassinosteroids

Brassinosteroids (BRs) are a class of polyhydroxylated steroidal phytohormones that play essential roles in regulating plant growth, development, and stress responses. The discovery of BRs dates back to the 1970s, when researchers isolated a growth promoting substance from pollen extracts of *Brassica napus*, later identified as brassinolide, the first biologically active brassinosteroid [6,7]. More than 70 naturally occurring BRs have been identified in various plant species, including monocots and dicots, demonstrating their widespread distribution and evolutionary conservation in the plant kingdom [8]. BRs are classified based on their structural characteristics into several groups, including C27, C28, and C29 BRs, depending on the number of carbon atoms in their steroid backbone. These compounds are synthesized from sterol precursors through a series of enzymatic reactions involving key biosynthetic enzymes such as DWARF4, Constitutive Photomorphogenesis and Dwarfism, and Brassinosteroid-6-oxidase [9,10]. The identification of these biosynthetic pathways has provided valuable insights into the molecular regulation of brassinosteroid production and function in plants.

### 1.4. Unique Feature of BRs Compared with Other Phytohormones

BRs possess several unique physiological and molecular characteristics that distinguish them from other plant hormones. Unlike many phytohormones that primarily regulate either growth or stress responses, BRs simultaneously control both developmental processes and stress tolerance mechanisms. They regulate diverse biological functions, including cell elongation, vascular differentiation, reproductive development, and senescence, while also enhancing plant resistance to environmental stresses [10,11]. One distinctive feature of BRs is their ability to modulate reactive oxygen species (ROS) homeostasis and the antioxidant defense system under stress conditions. BRs have been shown to stimulate the activity of antioxidant enzymes such as superoxide dismutase, catalase, and peroxidase, thereby protecting cellular components from oxidative damage [10,12]. Furthermore, BRs interact extensively with other phytohormones, including ABA, auxins, and ethylene, to regulate plant growth and stress adaptation through an integrated signaling network. This multifunctional nature makes BRs key regulators of plant resilience under adverse environmental conditions.

### 1.5. Scope and Objectives of the Review

Given the increasing challenges posed by environmental stresses and the growing demand for sustainable crop production, there is a pressing need to understand the regulatory mechanisms that enhance plant stress tolerance. BRs have emerged as critical components of plant stress signaling networks, yet many aspects of their roles in stress perception, signal transduction, and physiological adaptation remain incompletely understood. Recent advances in molecular biology, genomics, and system biology have provided new insights into the complex interactions between BRs and other signaling pathways involved in plant stress responses.

Although several recent reviews have discussed individual aspects of BR biology, including biosynthesis, signaling, hormone crosstalk, or specific stress responses, a comprehensive synthesis that integrates these processes into a unified framework explaining how BRs coordinate plant resilience across both biotic and abiotic stresses remains limited. Moreover, rapid advances in molecular genetics, multi-omics technologies, and genome-editing approaches have substantially expanded our understanding of BR-mediated regulatory networks and their potential applications for crop improvement. This review therefore provides a critical synthesis of recent findings by integrating BR biosynthesis, signaling, hormone crosstalk, ORS homeostasis, and stress-adaptive mechanisms into a mechanistic perspective of BR-mediated resilience. In addition, we discuss current limitations, emerging research directions, and opportunities for translating BR biology into breeding strategies for developing climate-resilient crops.

## 2. Literature Search Strategy

This review was prepared as a narrative review to provide a comprehensive synthesis of current knowledge on brassinosteroid in plant growth and stress responses. Relevant literature was identified through search of the Web of Science, Scopus, PubMed, and Google Scholar databases using combinations of keywords including brassinosteroid, BR signaling, BR biosynthesis, abiotic stress, biotic stress, phytohormones crosstalk, ROS, plant immunity, crop improvement, and stress tolerance. The literature search primarily covered publications from 2000 to 2026, while landmark studies describing the discovery of brassinsteroids and elucidation of their biosynthesis and signaling pathways were also included where appropriate. Preference was given to peer-reviewed research articles published in high-quality journals, together with recent review articles that provided broad context. Studies were selected based on their relevance to the scope of this review, focusing on molecular mechanisms, physiological functions, hormone interactions, and the application of brassinosteroids for improving plant resilience under biotic and abiotic stresses.

## 3. Brassinosteroid Biosynthesis, Metabolism, and Transport

Brassinosteroids are synthesized from sterol precursors through a tightly regulated network of enzymatic reactions that determine the production of biologically active molecules essential for plant growth and stress adaptation. The biosynthesis, catabolism, and distribution of BRs collectively maintain hormonal homeostasis and ensure proper physiological responses to environmental stimuli. Recent advances in molecular genetics and biochemical studies have significantly improved our understanding of the regulatory mechanisms controlling BR metabolism and transport in plants. These processes are highly dynamic and responsive to developmental signals as well as environmental stress conditions, allowing plants to fine-tune hormone levels according to physiological demands [13].

### 3.1. BRs Biosynthesis Pathways (Early and Late C-6 Oxidation Pathways)

**Figure 1:**
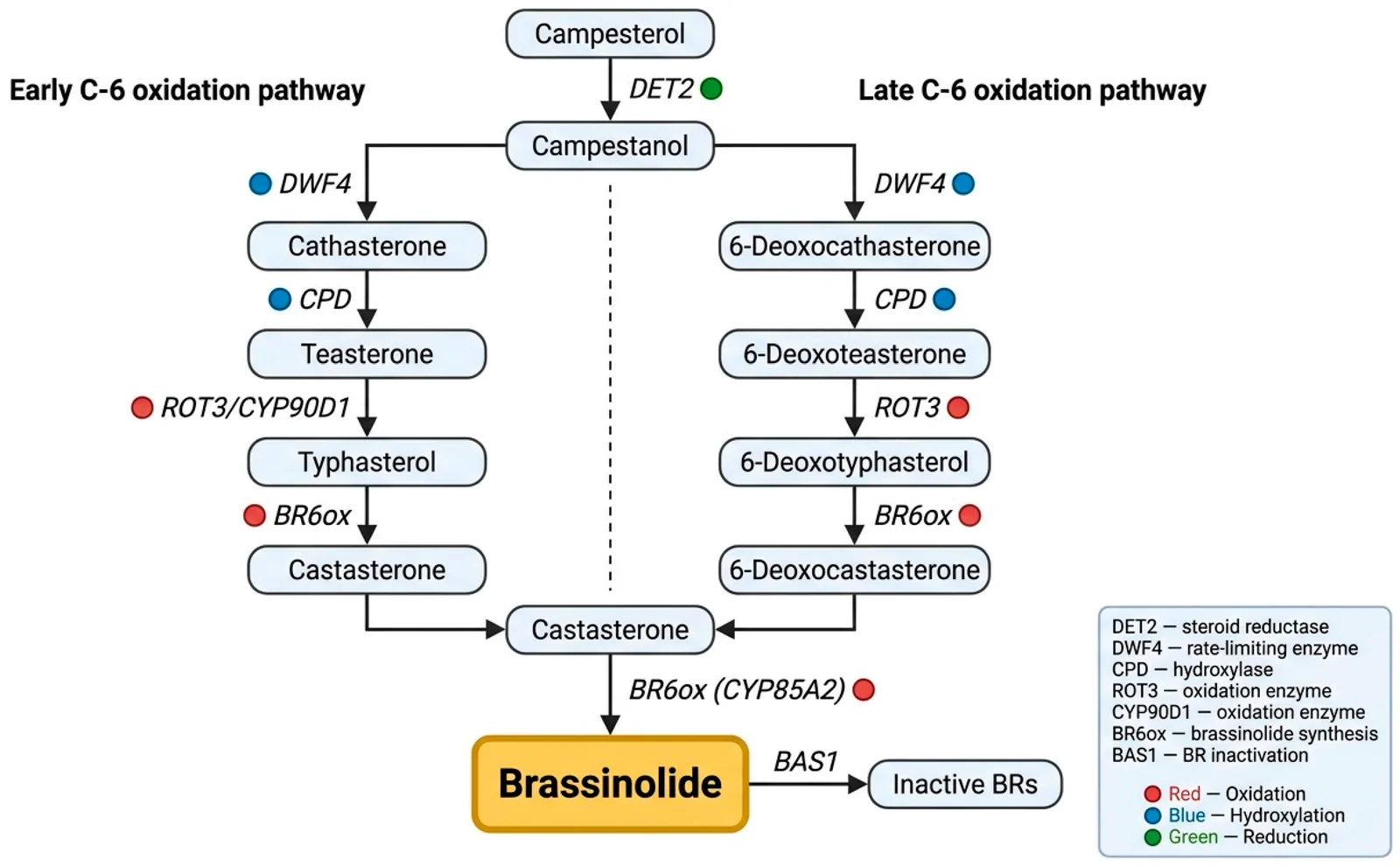
Brassinosteroid biosynthesis pathway in higher plants.

BRs biosynthesis originates from plant sterols, primarily campesterol, which undergoes multiple oxidation, reduction, and hydroxylation reactions to generate biologically active BRs such as castasterone and brassinolide (Figure 1). Two major parallel biosynthesis routes have been identified in higher plants, the early C-6 oxidation pathway and the late C-6 oxidation pathway, both of which ultimately converge to produce the active end products [9]. The biosynthetic sequence begins with the conversion of campesterol into campestanol through the action of the enzyme DF-ETIOLATED2 (DET2), a steroid reductase that catalyzes the reduction of the ∆5 double bond in sterol intermediates [9]. Campestanol then undergoes sequential hydroxylation and oxidation reactions mediated by cytochrome P450 monooxygenases, including DWARF4 (DWF4) and CONSTITUTIVE PHOTOMORPHOGENESIS AND DWARFISM (CPD), which introduce hydroxyl groups at specific carbons on the steroid backbone [14,15].

In the early C-6 oxidation pathway, oxidation at the C-6 position occurs before hydroxylation at the C-22 position. This route leads to the formation of intermediates such as cathasterone, teasterone, and typhasterol, which are subsequently converted into castasterone and finally into brassinolide, the most biologically active brassinosteroid [10,16]. In contrast, the late C-6 oxidation pathway involves hydroxylation at the C-22 position prior to C-6 oxidation, producing deoxy intermediates such as 6-deoxocathasterone and 6-deoxoteasterone before converging with the early pathway at the level of castasterone [17]. Current evidence indicates that the late C-6 oxidation pathway is frequently reported as the principal biosynthetic route in several model plant species; however, its relative predominance is species- and tissue-dependent and may vary during plant development [10,18]. The existence of multiple biosynthetic routes provides metabolic flexibility, enabling plants to maintain stable BR levels under diverse environmental conditions. Furthermore, feedback regulation mechanisms ensure that excessive accumulation of BRs is prevented, thereby maintaining hormonal balance within plant tissues [19].

### 3.2. Key Enzymes Involved in Brassinosteroid Biosynthesis

BRs biosynthesis is mediated by a group of enzymes that catalyze specific reactions at distinct stages of the pathway. Most of these enzymes belong to the cytochrome P450 superfamily, which plays a central role in steroid metabolism in plants. Among these enzymes, DWF4 is widely regarded as a rate-limiting enzyme because it controls the C-22 hydroxylation step, a critical regulatory point in the pathway [13]. Another key enzyme, CPD (CYP90A1), catalyzes hydroxylation at the C-23 position and is essential for the formation of downstream intermediates required for the synthesis of active BRs [20,21]. Similarly, ROTUNDIFOLIA3 (ROT3) and CYP90D1 are involved in the oxidation of intermediate compounds during the later stages of the pathway, contributing to the production of castasterone [20]. The final step in BR biosynthesis is catalyzed by BRASSINOSTEROID-6-OXIDASE (BR6ox), which converts castasterone into brassinolide. This step determines the biological activity of the hormone, as brassinolide exhibits the highest physiological potency among known brassinosteroids. Mutations in genes encoding these enzymes often result in dwarf phenotypes, delayed flowering, and reduced stress tolerance, demonstrating the critical role of BR biosynthetic enzymes in plant development [22].

### 3.3. Regulation of Brassinosteroid Biosynthesis Under Stress Conditions

The production of BRs is highly responsive to environmental stress signals. Abiotic stresses such as drought, salinity, and temperature extremes can significantly alter the expression of BR biosynthetic genes, thereby modifying endogenous hormone levels and activating stress adaptation mechanisms [12]. For instance, drought stress has been shown to induce the expression of DWF4 and CPD, leading to increased BR accumulation and improved water-use efficiency in plants. Transcription factors also play an important role in regulating BR biosynthesis under stress conditions. Proteins such as TCP1, ARF6, and BES1 have been reported to regulate the transcription of BR biosynthetic genes in response to environmental cues, thereby integrating hormonal signaling with stress responses [12,23]. Additionally, environmental factors such as light intensity, temperature, and nutrient availability influence BR biosynthesis through both transcriptional and post-translational mechanisms [8,24,25]. These regulatory processes enable plants to dynamically adjust BR levels according to environmental conditions, thereby enhancing stress tolerance and maintaining growth under advances condition.

### 3.4. Brassinosteroid Catabolism and Homeostasis

The maintenance of optimal BR concentrations within plant tissues depends on the balance between biosynthesis and catabolism. BR catabolism involves enzymatic modifications that convert active hormones into inactive or less active forms, thereby preventing excessive hormonal accumulation. One of the most important enzymes involved in BR catabolism is PHYB ACTIVATION TAGGED SUPPRESSOR 1 (BAS1), which catalyzes the hydroxylation of brassinolide and reduces its biological activity [20,26]. Other catabolic processes include glycosylation and conjugation reactions that facilitate hormone storage or degradation. These processes contribute to the maintenance of hormonal homeostasis and ensure that BR catabolic pathways can lead to abnormal growth patterns, reduced fertility, and altered stress responses, highlighting the importance of precise hormonal regulation in plant physiology.

### 3.5. Tissue-Specific Biosynthesis and Transport Mechanism

BR biosynthesis occurs in multiple plant tissues, including roots, shoots, leaves, and reproductive organs. However, the expression of biosynthetic genes is often spatially regulated, reflecting the diverse roles of BRs in plant development. For instance, higher expression levels of DWF4 and CPD have been observed in rapidly growing tissues such as young leaves and elongating stems, where BRs promote cell expansion and differentiation [27]. Although BRs are generally considered to act locally near their site of synthesis, recent studies have suggested that limited transport of BRs may occur between tissues through membrane transport proteins. Members of the ATP-binding cassette (ABC) transporter family have been implicated in the movement of steroid hormones across cellular membranes, thereby facilitating hormone distribution within plant tissues [28]. Nevertheless, the extent of long-distance transport of BRs remains an active area of research, and localized biosynthesis is still considered the primary mechanism controlling hormone availability in plants. Brassinosteroid homeostasis represents the first regulatory layer through which plants adjust hormone availability under environmental stress conditions. Stress signals such as drought, salinity, temperature fluctuations, and oxidative stress can modulate the expression and activity of BR biosynthetic enzymes, including DWF4, CPD, and BR6ox, thereby altering endogenous BR levels. Increased BT biosynthesis enhances the availability of active BR molecules, which are subsequently perceived by the BR1 receptor complex to initiate downstream signaling cascades. Conversely, activation of catabolic pathways mediated by enzymes such as BAS1 contributes to the attenuation of BR responses when excessive signaling may negatively affect growth-stress balance. Therefore, dynamic regulation of BR biosynthesis and degradation serves as an upstream control mechanism that determines the intensity and duration of BR signaling during stress adaptation. This metabolic regulation provides a mechanistic connection between environmental perception and downstream physiological responses by BR-responsive transcription factors.

Major BRs biosynthesis and metabolic genes and their functions are presented in Table 1.

## 4. Brassinosteroid Signaling Pathway

Brassinosteroid (BR)-mediated abiotic stress tolerance is achieved through a hierarchical regulatory framework that integrates hormone homeostasis, receptor-mediated signal transduction, transcriptional reprogramming, and downstream physiological adaptation. Environmental stresses, including drought, salinity, extreme temperatures, heavy metals, and oxidative stress, alter endogenous BR homeostasis by modulating the expression of BR biosynthetic and catabolic genes such as DWF4, CPD, BR6ox, and BAS1 [35,36]. Changes in active BR levels regulate the activation of the plasma membrane receptor BRI1 and its co-receptor BAK1, initiating a phosphorylation cascade involving BR-signaling kinases (BSKs), BSU1 phosphatase, and the negative regulator BIN2 [35,36]. Inactivation of BIN2 promotes the nuclear accumulation of the transcription factors BZR1 and BES1, which coordinate the expression of genes involved in reactive oxygen species (ROS) detoxification, osmotic adjustment, ion transport, photosynthetic protection, and stress-responsive metabolism. Through this coordinated regulatory network, BR signaling links environmental stress perception to cellular protection, ultimately improving physiological performance, maintaining growth, and enhancing crop productivity under adverse environmental conditions.

### 4.1. BR Perception by BRI1 and Co-Receptors (BAK1, SERK)

BR perception occurs at the plasma membrane through the receptor kinase BRASSINOSTEROID INSENSITIVE 1 (BRI1), which belongs to the leucine-rich repeat receptor-like kinases (LRR-RLK) family. BRI1 serves as the primary receptor responsible for recognizing brassnosteroids and initiating downstream signaling events [7]. In the absence of BRs, the receptor is maintained in an inactive state by the inhibitory protein BRI1 KINASE INHIBITOR 1 (BKI1), which prevents receptor activation and downstream signal propagation [7,37]. Upon binding of biologically active BRs such as brassinolide, conformational changes in the extracellular domain of BRI1 promote its association with co-receptors from the SOMATIC EMBRYOGENESIS RECEPTOR KINASE (SERK) family. Among these, BRI1-ASSOCIATED RECEPTOR KINASE 1 (BAK1) plays a central role in stabilizing the receptor complex and facilitating signal initiation [38]. The formation of the BRI1-BAK1 heterodimer triggers reciprocal transphosphorylation of their intracellular kinase domains, leading to activation of downstream signaling components [38]. Recent studies have demonstrated that additional SERK members, including SERK1 and SERK4, contribute to receptor complex formation and signaling efficiency, particularly under stress conditions. These co-receptors enhance receptor sensitivity and contribute to the robustness of BR perception in plants exposed to environmental challenges [39].

### 4.2. Signal Transduction Cascade (BSKS, BIN2, BSU1)

Following receptor activation, BR signals are transmitted into the cytoplasm through a phosphorylation cascade involving multiple signaling intermediates. One of the earliest events in this process is the phosphorylation of BR-SIGNALING KINASES (BSKs) by activated BRI1, which enables the relay of signals from the plasma membrane to downstream regulatory proteins [38]. Activated BSKs interact with the phosphatase BRI1-SUPPRESSOR 1 (BSU1), which plays a crucial role in regulating the activity of negative regulators of BR signaling. A key target of BSU1 is BRASSINOSTEROID INSENSITIVE 2 (BIN2), a glycogen synthase kinase-3 (GSK3)-like protein that functions as a central inhibitor of the pathway [38,40]. In the absence of brassinosteroid, BIN2 remains active and phosphorylates downstream transcription factors, thereby preventing their activation and nuclear accumulation [38]. When brassinosteroids are present, BSU1-mediated dephosphorylation inactivates BIN2, resulting in the release of transcription factors from inhibitory phosphorylation [38,40]. This transition from an inactive to an active signaling state represents a key regulatory checkpoint controlling plant growth and stress responses.

### 4.3. Transcriptional Regulation by BZR1 and BES1

The final stage of BR signaling involves transcriptional regulation of BR-responsive genes by the transcription factors BRASSINAZOLE-RESISTANT 1 (BZR1) and BRI1-EMS-SUPPRESSOR 1 (BES1). These transcription factors function as master regulators of gene expression controlling cell expansion, vascular development, and stress tolerance [41,42]. In the absence of BRs, BZR1 and BES1 are phosphorylated by BIN2, which results in their retention in the cytoplasm mediated by 14-3-3 protein binding, and subsequent degradation via the ubiquitin-proteasome pathway [43]. However, when BIN2 activity is suppressed following BR perception, these transcription factors become dephosphorylated and accumulate in the nucleus, where they bind to promoter regions of BR-responsive genes and regulate transcription [38]. BZR1 and BES1 bind to target promoters containing BRRE (BR-response element; CGTGC/TG) and/or E-box (CANNTG) motifs to regulate the expression of thousands BR-responsive genes that are crucial for plant growth and development [38].

### 4.4. Stress-Dependent Modulation of BR Signaling

BR signaling is strongly influenced by environmental stress conditions, enabling plants to adjust growth and defense responses accordingly. Abiotic stresses such as drought, salinity, temperature extremes, and heavy metal toxicity have been shown to modulate the expression and activity of BR signaling components [12]. For instance, drought stress can alter the phosphorylation status of BIN2 and related kinases, leading to changes in transcription factor activity and gene expression patterns associated with stress tolerance [44]. Similarly, salinity stress has been reported to influence the expression of BR receptor genes and downstream signaling components, thereby enhancing antioxidant defense mechanisms and osmotic adjustment in plants [12,45,46]. Recent evidence also indicated that BR signaling interacts extensively with other plant hormone pathways, including ABA, ethylene, and JA signaling networks. This hormonal crosstalk enables plants to balance growth and defense responses under stress conditions [45].

### 4.5. Post-Translational Regulation of BR Signaling Components

**Figure 2:**
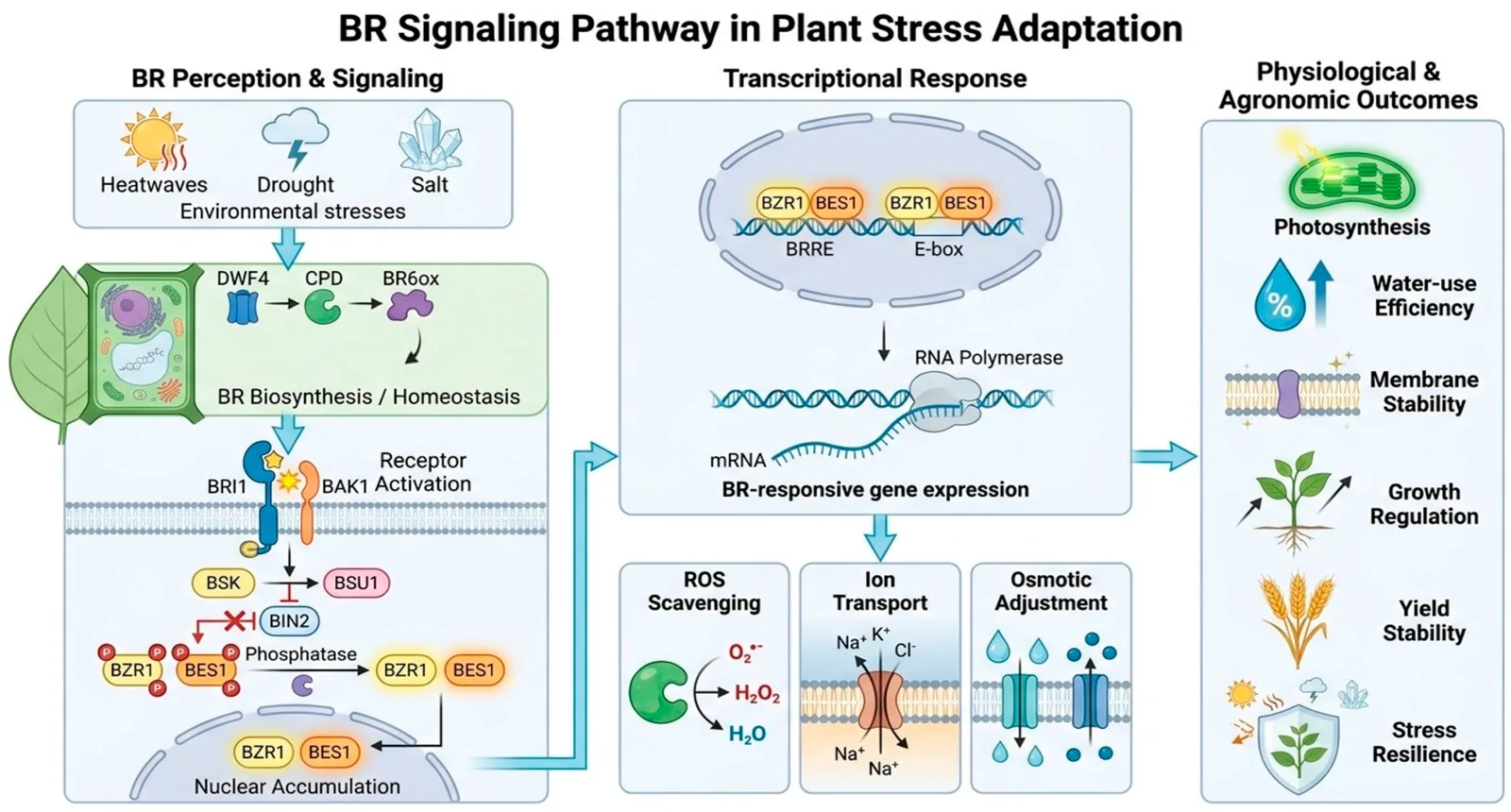
Schematic representation of the brassinosteroid (BR) signaling pathway and its role in plant responses to environmental stresses. Environmental stresses, including heat, drought, and salinity, can influence BR biosynthesis and homeostasis through BR biosynthetic components such as DWF4, CPD, and BR6ox. BR perception by the BRI1–BAK1 receptor complex initiates downstream signaling through BSK and BSU1, leading to inhibition of the GSK3-like kinase BIN2. Reduced BIN2 activity decreases the phosphorylation of the transcription factors BZR1 and BES1, allowing their dephosphorylation, nuclear accumulation, and binding to BR-responsive elements (BRREs) and E-box sequences. Activated BZR1/BES1 promote the transcription of BR-responsive genes involved in reactive oxygen species (ROS) scavenging, ion transport, and osmotic adjustment. Collectively, these responses contribute to the maintenance of photosynthetic activity, water-use efficiency, membrane stability, growth regulation, yield stability, and overall stress resilience. Arrows indicate positive or sequential regulatory events, whereas blunt-ended lines indicate inhibitory interactions.

Post-translational modifications play a critical role in regulating BR signaling and ensuring appropriate cellular responses to hormonal signals. These modifications include phosphorylation, ubiquitination, SUMOylation, and proteasome-mediated degradation of signaling proteins [47]. Phosphorylation is particularly important for controlling the activity and localization of transcription factors such as BZR1 and BES1. Following BR perception by the BRI1–BAK1 receptor complex, downstream signaling through BSK1 and BSU1 suppresses the activity of the GSK3-like kinase BIN2. In the absence of BR signaling, BIN2-mediated phosphorylation keeps BZR1 and BES1 predominantly inactive and promotes their cytoplasmic retention, whereas BR-induced BIN2 inhibition facilitates their dephosphorylation and subsequent accumulation in the nucleus. The phosphorylation state of these proteins determines whether they remain inactive in the cytoplasm or translocate to the nucleus to regulate gene expression [47]. In addition to phosphorylation, ubiquitination and SUMOylation provide additional layers of post-translational control by modulating the stability, activity, and subcellular localization of BR signaling components. E3 ubiquitin ligases can target selected signaling proteins for ubiquitination and subsequent proteasome-mediated degradation, thereby preventing excessive or prolonged BR responses and maintaining signaling homeostasis [47]. Together, these reversible and irreversible post-translational mechanisms provide fine control over the abundance, localization, and transcriptional activity of BR signaling components. Collectively, the processes described in Section 2 and Section 3 illustrate a hierarchical regulatory framework in which environmental stresses modulate BR homeostasis through changes in biosynthesis and catabolism, leading to receptor-mediated signal transduction, post-translational regulation of key signaling components, including phosphorylation/dephosphorylation and protein turnover, transcriptional reprogramming, hormonal crosstalk, and physiological adaptations that ultimately improve plant performance under adverse conditions. This integrated mechanism is summarized in Figure 2.

## 5. Role of BR in Abiotic Stress Tolerance

Brassinosteroids (BRs) regulate abiotic stress tolerance through a hierarchical network involving both conserved stress-protective mechanisms and stress-specific adaptive responses. Common BR-associated outputs across diverse environmental stresses include regulation of reactive oxygen species (ROS) homeostasis, maintenance of membrane stability, osmotic adjustment, metabolic reprogramming, and activation of stress-responsive transcriptional networks. BR-mediated activation of antioxidant systems, metabolic adjustment, and stress-responsive genes represents a conserved mechanism contributing to cellular protection under unfavorable environmental conditions [48]. However, the downstream consequences of BR signaling differ depending on the nature of the stress. During drought stress, BRs primarily contribute to adaptive responses through regulation of stomatal behavior, water-use efficiency, and interaction with ABA-dependent signaling pathways. Under salinity conditions, BR-mediated tolerance is associated with maintenance of Na^+^/K^+^ homeostasis and regulation of ion transport processes. Temperature stress responses involve BR-dependent regulation of heat shock proteins, cold acclimation pathways, and membrane stability, whereas heavy metal tolerance involves metal chelation, sequestration, and detoxification mechanisms.

### 5.1. Drought Stress

#### 5.1.1. Regulation of Stomatal Movement and Water-Use Efficiency

Drought stress is one of the most critical environmental factors limiting plant growth and crop productivity worldwide. BRs have been shown to regulate stomatal behavior and improve water-use efficiency by modulating guard cell signaling pathways and maintaining leaf water status. Under moderate drought stress, BR treatment often promotes partial rather than complete stomatal closure, thereby reducing excessive transpiration while allowing sufficient CO_2_ uptake to sustain photosynthesis [10,49]. Consequently, BR-mediated improvement in water-use efficiency reflects optimization of the balance between water conservation and carbon assimilation rather than maximum stomatal closure [49]. In addition to stomatal regulation, BRs help maintain photosynthetic capacity through non-stomatal mechanisms, including protection of chloroplast integrity and preservation of photosynthetic enzyme activity under drought conditions. However, the magnitude of these responses varies among plant species, developmental stages, BR concentrations, and stress severity, indicating that optimal BR-mediated drought tolerance requires species- and condition-specific regulation.

#### 5.1.2. BR-Mediated Antioxidant Defense and Osmolyte Accumulation

Under drought stress, plants experience oxidative damage due to excessive accumulation of reactive oxygen species. BRs enhance the activity of antioxidant enzymes such as SOD, CAT, POD, thereby reducing oxidative stress and protecting cellular components. In addition, BRs promote the accumulation of osmolytes such as proline, soluble sugars, and glycine betaine, which help maintain cellular osmotic balance and protect proteins and membranes under dehydration conditions [50,51,52].

#### 5.1.3. Interaction with ABA Signaling

BRs also interact with abscisic acid (ABA), a central regulator of drought responses, to coordinate growth, stomatal regulation, and stress adaptation [53,54]. Because the molecular mechanisms and physiological significance of BR–ABA crosstalk are discussed in detail in Section 7, this section only highlights its relevance to the broader role of BRs in abiotic stress responses.

### 5.2. Salinity Stress

#### 5.2.1. Maintenance of Ion Homeostasis (Na^+^/K^+^ Balance)

Salinity stress disrupts cellular ion balance, leading to excessive accumulation of sodium ion and reduced potassium uptake. BRs play a crucial role in maintaining ion homeostasis by regulating ion transporters and enhancing selective uptake of potassium ions. This mechanism helps maintain the Na^+^/K^+^ ratio, which is essential for enzyme activity, osmotic regulation, and cellular stability under saline conditions [12,55].

#### 5.2.2. Protection of Membrane Integrity

High salinity causes membrane damage due to lipid peroxidation and oxidative stress. BRs improve membrane stability by enhancing the antioxidant defense system and reducing the accumulation of toxic reactive oxygen species. This protective effect helps maintain membrane permeability and cellular integrity under salt stress conditions [55,56].

#### 5.2.3. BR Regulation of Stress Responsive Genes

BRs regulate the expression of stress-responsive genes involved in ion transport, osmotic adjustment, and antioxidant defense. Transcriptomic studies have shown that BR signaling activates genes encoding ion transporters, transcription factors, and stress-protective proteins, thereby enhancing plants tolerance to salinity stress [57].

### 5.3. Temperature Stress

Exposure to high temperatures causes protein denaturation, membrane instability, disruption of photosynthetic processes, and excessive reactive oxygen species (ROS) accumulation. BRs contribute to thermotolerance through multiple regulatory mechanisms, including modulation of heat shock proteins (HSPs), antioxidant systems, photosynthetic protection, and stress-responsive signaling pathways [58]. However, the magnitude of BR-mediated temperature tolerance depends on plant species, developmental stage, stress intensity, and whether responses are induced through exogenous application or endogenous regulation of BR signaling. Additionally, BRs stimulate antioxidant enzymes that detoxify reactive oxygen species and protect cellular structure from thermal damage [59]. Similarly, low temperature stress affects membrane fluidity and metabolic processes, leading to reduced growth and cellular injury. BRs enhance cold tolerance by maintaining membrane stability, improving photosynthetic efficiency, and regulating the expression of cold-responsive genes involved in stress adaptation [60]. These mechanisms enable plants to maintain metabolic activity and survive under low temperature conditions. BR responses to temperature stress involve both conserved and stress-specific regulatory pathways. Under heat stress, BR signaling components such as BIN2, BZR1, and BES1 participate in transcriptional regulation of heat-responsive genes, while BR-mediated activation of antioxidant defenses and photoprotective mechanisms helps maintain cellular homeostasis [49,61]. Under chilling and freezing conditions, BRs influence cold acclimation through interactions with CBF-dependent and CBF-independent pathways, although the precise mechanisms remain incompletely understood [49,62]. In addition, BR-mediated regulation of autophagy, membrane remodeling, and energy metabolism contributes to adaptation under prolonged temperature stress [63]. Importantly, temperature responses vary among species; while many mechanistic studies have been performed in Arabidopsis, crop studies indicate that BR effects on temperature tolerance are strongly influenced by genotype, developmental stage, and environmental conditions. Therefore, understanding how endogenous BR signaling and exogenous BR treatments regulate temperature adaptation in diverse crops remains an important research priority.

### 5.4. Heavy Metal and Toxic Element Stress

Heavy metals such as aluminum, cadmium, and lead cause toxicity in plants by disrupting cellular metabolism, generating oxidative stress, and inhibiting growth. BRs have been shown to reduce heavy metal toxicity by enhancing antioxidant activity, regulating metal transport, and improving cellular detoxification processes [64,65]. Similarly, BRs stimulate the production of metal-binding compounds such as phytochelatins and metallothioneins, which chelate toxic metal ions and reduce their harmful effects [66]. Additionally, BRs enhance the activity of detoxification and antioxidant enzymes, thereby reducing oxidative damage and protecting cellular structure under heavy metal stress [65,67].

### 5.5. Oxidative Stress

Oxidative stress occurs when the production of reactive oxygen species exceeds the capacity of cellular antioxidant systems. Under abiotic stress, BRs enhance ROS scavenging by activating enzymatic and non-enzymatic antioxidant systems, thereby preventing the excessive accumulation of damaging ROS while maintaining the basal ROS levels required for normal cellular signaling. These protective mechanisms are essential for maintaining cellular homeostasis under various abiotic stress conditions. Likewise, BRs regulate the expression and activity of key antioxidant enzymes, including SOD, CAT, POD, and glutathione reductase [68]. Increased activity of these enzymes enhances stress tolerance by reducing oxidative damage to proteins, lipids, and nucleic acids. Although BRs are widely recognized for enhancing antioxidant capacity and limiting excessive ROS accumulation under abiotic stress, their effects on ROS are context-dependent rather than uniformly suppressive. BR signaling regulates redox homeostasis by balancing ROS production and scavenging according to the type, intensity, duration, and spatial distribution of stress. During abiotic stress, prolonged or excessive ROS accumulation causes oxidative damage to proteins, lipids, and nucleic acids; therefore, BRs enhance antioxidant defenses to restore cellular redox balance. In contrast, during biotic stress, BR signaling promotes a rapid and localized ROS burst at infection sites, where ROS functions as an early signaling molecule that activates immune responses and contributes to pathogen restriction. Subsequently, antioxidant systems confine ROS accumulation to prevent collateral damage to host tissue. Thus, BR-mediated regulation of ROS represents a dynamic spatiotemporal redox homeostasis mechanism that coordinates ROS signaling with antioxidant-mediated detoxification, enabling plants to balance stress adaptation, immune defense, and cellular survival.

## 6. Role of BRs in Biotic Stress Resistance

**Figure 3:**
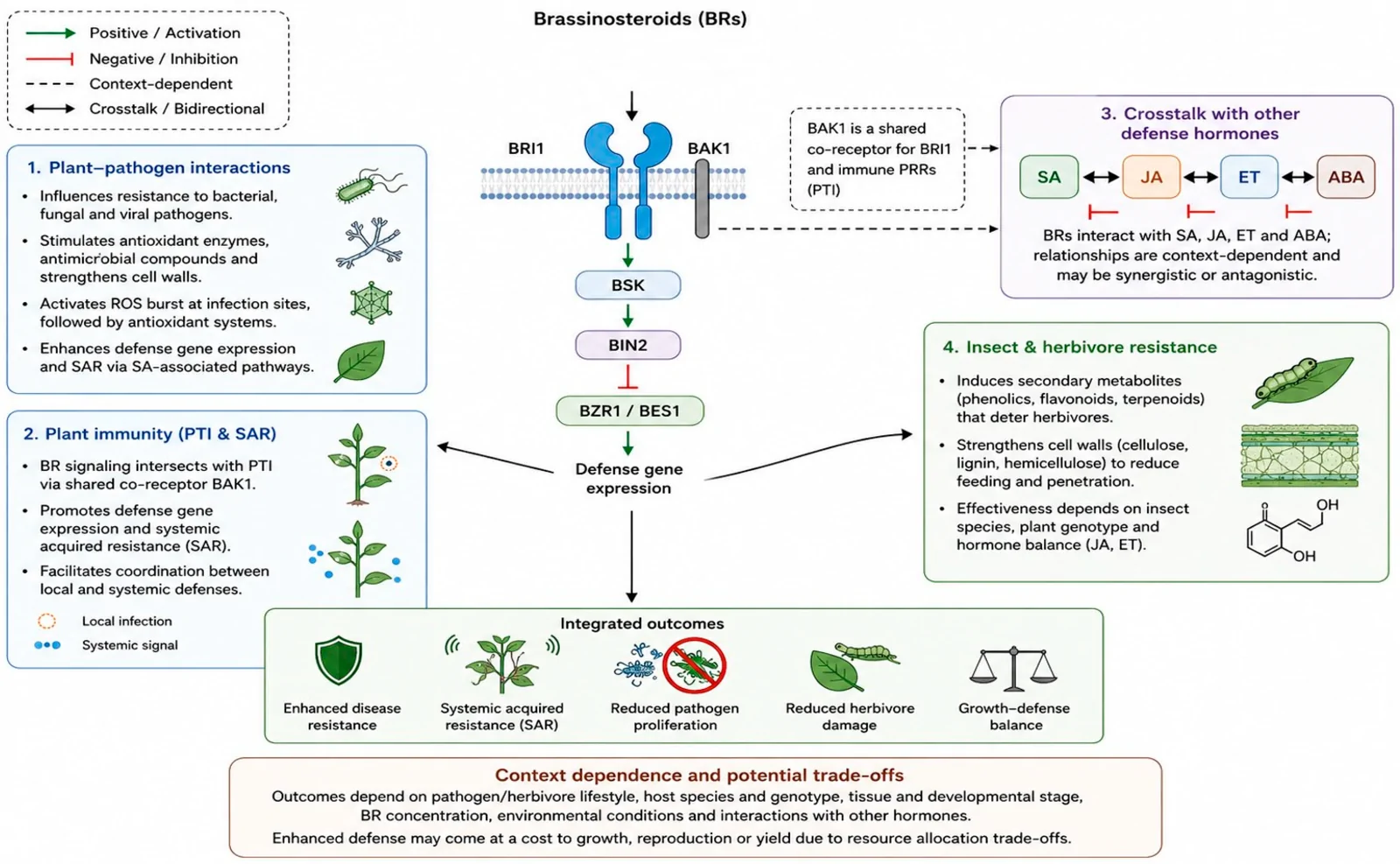
Role of brassinosteroids (BRs) in biotic stress resistance. BR signaling is initiated by BR perception through the BRI1–BAK1 receptor complex and proceeds through BSK and BIN2 to regulate BZR1/BES1 activity and defense-related gene expression. The inhibitory effect of BIN2 on BZR1/BES1 is indicated by a bar-ended line. BR signaling intersects with pattern-triggered immunity (PTI) through the shared co-receptor BAK1 and contributes to systemic acquired resistance (SAR), pathogen resistance, and herbivore resistance through regulation of defense-related genes, reactive oxygen species (ROS), cell-wall strengthening, and antimicrobial/secondary metabolite accumulation. Crosstalk with salicylic acid (SA), jasmonic acid (JA), ethylene (ET), and abscisic acid (ABA) is shown as context-dependent and may involve positive, negative, or bidirectional interactions. Overall, BR-mediated biotic stress responses depend on pathogen or herbivore lifestyle, plant genotype, tissue and developmental stage, BR concentration, environmental conditions, and stress severity, and may involve trade-offs between defense and growth, reproduction, or yield. Arrowheads indicate activation or positive regulation, bar-ended lines indicate inhibition, bidirectional arrows indicate reciprocal or complex interactions, and dashed lines indicate context-dependent or variable relationships.

BRs play a significant role in regulating plant defense responses against a wide range of biotic stresses, including bacterial, fungal, and viral pathogens, and herbivorous insects [69]. These phytohormones modulate immune signaling pathways, regulate defense gene expression, and coordinate physiological responses that enhance plant resistance while maintaining growth and development. Increasing evidence indicates that BR signaling interacts extensively with plant immune pathways, including pattern-triggered immunity (PTI) and systemic acquired resistance (SAR), thereby enabling plants to respond effectively to pathogen invasion [64,70,71]. BR-mediated defense responses involve activation of antioxidant enzymes, reinforcement of cell walls, accumulation of antimicrobial compounds, and regulation of defense-related gene expression [72]. These mechanisms collectively contribute to enhanced plant immunity and improved resistance to pathogens and herbivores under stress conditions. This integrated mechanism is summarized in Figure 3. However, BR-mediated regulation of biotic stress resistance is highly context-dependent and does not always result in enhanced immunity. The outcome of BR signaling depends on pathogen lifestyle, host species, tissue type, developmental stage, BR concentration, and interactions with other defense hormones, particularly SA, JA, and ethylene. Evidence from exogenous BR applications, BR-deficient mutants, BR-insensitive mutants, and genetic activation of BR signaling indicates that BRs can either promote or suppress immune responses depending on the biological context. Therefore, BR signaling should be considered as a regulatory mechanism that balances growth and defense rather than as a universal positive regulator of plant immunity.

### 6.1. BRs in Plant-Pathogen Interactions

Plants encounter diverse microbial pathogens that can significantly reduce crop productivity. BRs have been reported to influence resistance against bacterial, fungal, and viral pathogens by modulating immune signaling pathways and strengthening physical barriers such as cell walls; however, the magnitude and direction of these effects vary depending on pathogen lifestyle, host genotype, and experimental conditions. For instance, exogenous BR treatments have reduced disease severity caused by bacterial pathogens such as *Pseudomonas syringae*, fungal pathogens including *Magnaporthe grisea*, and viral infections in several plant species [73,74]. However, these responses may not always reflect endogenous BR functions, as BR-deficient or BR-insensitive mutants and plants with genetically enhanced BR signaling can exhibit distinct immune phenotypes depending on the pathogen and host system. BR-induced resistance is associated with increased production of antimicrobial compounds, activation of defense enzymes, and improved cellular integrity [69]. These responses help limit pathogen growth and spread within plant tissues, thereby enhancing plant survival under pathogen attack. BRs regulate the expression of numerous defense-related genes involved in pathogen resistance [53]. These genes encode pathogenesis-related proteins, antimicrobial peptides, and enzymes involved in secondary metabolite biosynthesis. Transcriptomic analyses have demonstrated that BR signaling activates transcription factors that control defense gene expression and enhance immune responses during pathogen infection [53]. In addition, BR signaling promotes a rapid and localized production of reactive oxygen species (ROS) at pathogen infection sites. This transient ROS burst functions as an early signaling event that activates downstream immune responses, strengthens cell walls, and contributes to pathogen restriction. Subsequently, antioxidant systems limit ROS diffusion to prevent excessive oxidative damage to surrounding host tissues [53].

Similarly, SAR is a long-lasting, broad-spectrum defense mechanism that provides protection against subsequent pathogen infections. BRs have been shown to enhance SAR by regulating signaling pathways associated with SA and defense gene expression. BR signaling can promote the accumulation of SAR-related proteins and activate systemic defense responses throughout the plant [69,74,75]. Recent studies indicate that BR-mediated SAR involves coordination between local immune responses and systemic signaling networks, enabling plants to establish long-term resistance against pathogens [69].

### 6.2. BRs and Plant Immunity

Pattern-triggered immunity (PTI) represents the first line of defense against pathogen invasion. This immune response is initiated by recognition of pathogen-associated molecular patterns (PAMPs) by pattern recognition receptors (PRRs) located on the plasma membrane. BR signaling intersects with plant immune pathways through shared signaling components, particularly the co-receptor BRI1-ASSOCIATED RECEPTOR KINASE 1 (BAK1). BAK1 functions as a common partner for both the BR receptor BRI1 and several pattern-recognition receptors (PRRs) involved in pattern-triggered immunity (PTI), allowing coordination between growth regulation and immune activation [76]. However, the utilization of BAK1 by multiple receptor complexes does not necessarily indicate that BR signaling always enhances immunity. Instead, BAK1 acts as a signaling hub whose activity is determined by receptor availability, ligand perception, developmental stage, and environmental context [77]. Competition for shared signaling components, differential receptor-complex formation, and pathway-specific phosphorylation events may influence the relative strength of BR- and immune-related responses. Therefore, BAK1-mediated integration of BR and immune signaling represents a dynamic regulatory mechanism rather than a simple additive relationship. Under some conditions, BR signaling may support immune responses by enhancing defense-associated transcriptional programs, whereas under other conditions, resource allocation toward growth or activation of competing pathways may limit immune outputs. This crosstalk enables plants to coordinate growth and defense responses under pathogen attack. The integration of BR signaling with immune receptors enhances the efficiency and specificity of defense responses. Recent molecular studies have shown that BR signaling can modulate immune receptor activity through phosphorylation and transcriptional regulation mechanisms, thereby influencing plant resistance to pathogens [69]. Importantly, studies using exogenous BR application, BR biosynthesis mutants, BR receptor mutants, and constitutive activation of BR signaling have revealed that the relationship between BR signaling intensity and immunity is not linear, with both enhanced and reduced resistance reported depending on genetic background and stress conditions. Plants must continuously balance growth and defense to optimize survival under changing environmental conditions. Because activation of immune responses requires substantial metabolic resources, enhanced defense is frequently accompanied by reduced vegetative growth or reproductive performance. Rather than eliminating this trade-off, BR signaling functions as a regulatory module that dynamically adjusts resource allocation between growth and immunity according to developmental stage, environmental conditions, and stress severity. Moderate BR signaling can improve stress resilience while maintaining growth; however, excessive or prolonged BR accumulation may promote stem elongation, alter plant architecture, or reduce reproductive fitness. Conversely, reduced BR signaling can enhance certain immune responses but is often associated with dwarfism and impaired development. Therefore, the biological outcome of BR signaling is context-dependent and reflects optimization of the growth–defense balance rather than simultaneous maximization of both processes.

### 6.3. BRs in Insect and Herbivore Resistance

Herbivorous insects cause significant damage to plants by feeding on leaves, stems, and reproductive tissues. BRs can contribute to insect herbivore resistance by promoting the synthesis of secondary metabolites such as phenolics, flavonoids, and terpenoids; however, the effectiveness of BR-mediated herbivore defense depends on insect species, plant genotype, and interactions with other defense hormones such as JA and ethylene [78,79]. These compounds function as chemical defenses that deter insect feeding and reduce herbivore damage. Increased accumulation of secondary metabolites improves plant defense capacity and contributes to reduced herbivore infestation in crop plants [80]. BRs strengthen plant cell walls by promoting the synthesis of structural components such as cellulose, lignin, and hemicellulose [81]. Enhanced cell wall thickness and mechanical strength provide physical barriers that reduce insect feeding and pathogen penetration [82]. Additionally, BR-mediated changes in cell architecture improve plant resistance to mechanical damage caused by herbivores and environmental stress factors. In natural environments, plants are frequently exposed to simultaneous biotic and abiotic stresses rather than isolated stress conditions. Therefore, BR-mediated responses under combined stresses cannot always be predicted based solely on single-stress studies. Interactions between different stress signals may involve shared, synergistic, antagonistic, or sequential regulatory mechanisms depending on stress type, intensity, duration, and developmental context. Under combined stress conditions, BR signaling may integrate multiple hormonal pathways, including ABA, SA, JA, and ethylene, to coordinate growth, defense, and stress adaptation. Understanding these context-dependent responses will be essential for accurately defining the contribution of BR signaling to plant resilience under realistic field environments.

## 7. Crosstalk Between BRs and Other Phytohormones

**Figure 4:**
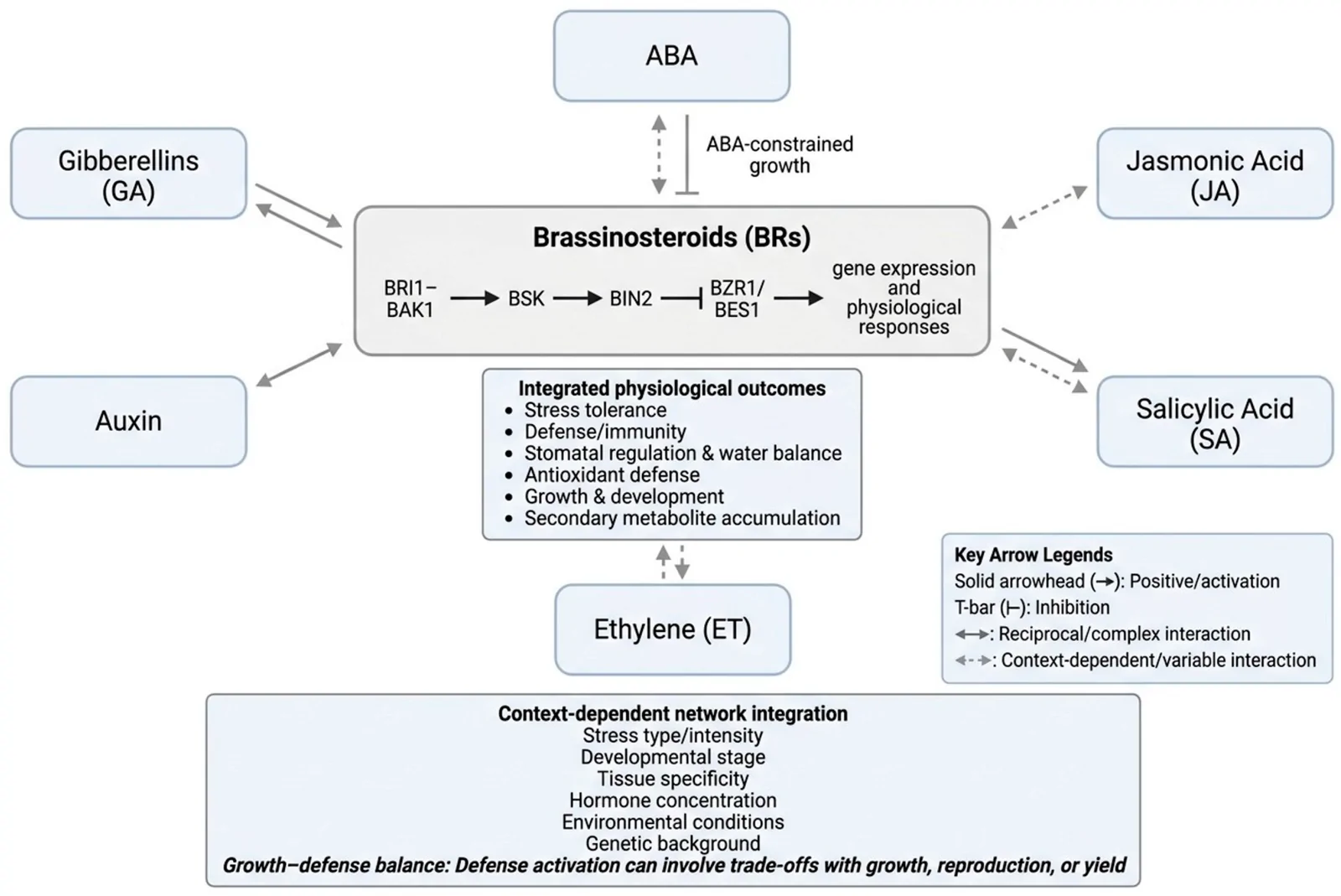
Crosstalk between brassinosteroids (BRs) and other phytohormones in the regulation of plant growth and stress responses. BR signaling interacts with abscisic acid (ABA), jasmonic acid (JA), salicylic acid (SA), ethylene (ET), auxin, and gibberellins (GA) at multiple regulatory levels, including hormone biosynthesis, signal transduction, transcriptional regulation, and downstream physiological responses. The core BR signaling pathway involves the BRI1–BAK1 receptor complex, BSK, BIN2, and BZR1/BES1, which regulate downstream gene expression. BR–ABA interactions contribute to stomatal regulation, water balance, osmotic adjustment, and stress-responsive gene expression, while ABA activity can also constrain BR-mediated growth. BR interactions with JA and SA contribute to defense-related gene expression, secondary metabolite production, systemic acquired resistance, and pathogen defense. BR–ethylene interactions regulate stress adaptation, growth inhibition, senescence, and developmental responses. BR–auxin and BR–GA interactions coordinate cell elongation, vascular differentiation, root development, plant architecture, biomass accumulation, and developmental transitions. The overall outcome of BR–hormone crosstalk is context-dependent and is influenced by stress type and intensity, developmental stage, tissue specificity, hormone concentration, environmental conditions, and genetic background. These interactions ultimately contribute to the balance between growth, defense, and stress adaptation and may involve trade-offs with growth, reproduction, or yield. Solid arrows indicate positive/activating interactions, T-bars indicate inhibitory interactions, double-headed arrows indicate reciprocal or complex interactions, and dashed double-headed arrows indicate context-dependent or variable interactions.

BRs function as central regulators of plant growth and stress responses, but they rarely act independently. Instead, BR signaling interacts extensively with other phytohormones and pathways, forming complex regulatory networks that coordinate plant development and adaptation to environmental stress. These interactions occur at multiple levels, including hormone biosynthesis, signal transduction, transcriptional regulation, and protein modification. Such network-level integration enables plants to optimize growth while maintaining resilience to abiotic and biotic stresses. Recent studies demonstrated that BR signaling interacts with several key hormones, including ABA, JA, SA, ethylene, auxin, and GA, to regulate stress tolerance, immune responses, and developmental processes [49,83]. These hormonal interactions can be synergistic or antagonistic depending on environmental conditions and developmental stage, allowing plants to fine-tune physiological responses to stress (Figure 4).

### 7.1. BR-ABA Interaction in Abiotic Stress

ABA is a major regulator of plant responses to abiotic stresses such as drought, salinity, and cold. BRs interact closely with ABA signaling pathways to regulate stomatal movement, osmotic balance, and stress-responsive gene expression [84]. This interaction plays a crucial role in maintaining cellular homeostasis and improving plant survival under adverse environmental conditions. Under drought stress, BR signaling enhances ABA sensitivity in guard cells, promoting stomatal closure and reducing water loss [84,85,86]. Additionally, BR-ABA interactions regulate the expression of genes involved in antioxidant defense, thereby improving plant tolerance to several stresses [49]. At the molecular level, BR and ABA pathways share common signaling components and transcription factors that coordinate stress responses. Although BR–ABA crosstalk can positively enhance drought tolerance through improved stomatal regulation and stress-responsive defense, increased ABA activity may also constrain BR-mediated growth promotion, illustrating the trade-off between stress protection and growth maintenance.

### 7.2. BR-JA and BR-SA Crosstalk in Biotic Stress

JA and SA are key hormones regulating plant immune responses against pathogens and herbivores. BRs interact with JA and SA signaling pathways to modulate defense responses and maintain the balance between growth and immunity [83,87]. These interactions are essential for coordinating plant responses to pathogen infection and insect attack. BR-JA crosstalk often regulates defense-related gene expression and secondary metabolite production, which enhances plant resistance to biotic stress [88]. For instance, JA signaling components such as MYC2 transcription factors interact with BR signaling pathways to integrate growth and defense responses [89]. Similarly, BR-SA interactions play a critical role in systemic acquired resistance (SAR) and pathogen defense [70]. These hormonal interactions regulate the expression of pathogen-related proteins and antimicrobial compounds, thereby enhancing plant immunity against bacterial and fungal pathogens. Thus, BR–JA–SA interactions can be positive when coordinated signaling enhances pathogen resistance, but can also be antagonistic when defense activation suppresses growth or when BR-mediated growth promotion attenuates excessive immune responses. Because BR signaling promotes growth while JA and SA frequently activate defense-associated metabolic programs, their interaction represents a balance between resource allocation toward growth and immunity. Depending on pathogen type, infection stage, and hormone concentration, BR signaling may enhance defense responses or attenuate immune activation to prevent excessive fitness costs.

### 7.3. BR-Ethylene Interaction During Stress Adaptation

Ethylene is an important hormone regulating plant growth, senescence, and stress adaptation. BRs interact with ethylene signaling pathways to regulate plant responses to environmental stress conditions such as salinity, flooding, and pathogen infection [83,90,91,92]. These interactions influence gene expression, hormonal balance, and physiological responses that enhance plant survival under stress conditions. For instance, ethylene signaling can modulate BR biosynthesis and signaling activity during stress adaptation, thereby influencing plant growth and defense responses [93,94]. This interaction occurs through regulation of BR biosynthetic genes, modulation of receptor signaling activity, and convergence of downstream transcription factors controlling stress adaptation. Thus, BR–ethylene interactions can be either synergistic or antagonistic depending on the physiological context, with positive interactions promoting stress adaptation and negative interactions contributing to growth inhibition, senescence, or developmental adjustment. This coordination regulation enables plants to adjust metabolic processes and maintain cellular homeostasis under adverse environmental conditions. In addition, BR-ethylene interactions regulate processes such as leaf senescence, root development, and stress-induced growth inhibition, highlighting the importance of hormonal coordination in plant stress physiology [90].

### 7.4. BR-Auxin and BR-GA Coordination in Growth-Stress Balance

Auxin and GA are major regulators of plant growth and development, and their interaction with BRs plays a central role in coordinating growth and stress responses. BR-auxin crosstalk regulates cell elongation, vascular differentiation, and root development through coordinated control of gene expression and hormone transport [95]. These interactions are particularly important for regulating plant architecture and biomass accumulation under stress conditions. For example, BR auxin signaling pathways cooperate to control cell division and elongation, which determine plant growth and productivity [95]. Similarly, BR-GA interactions regulate growth-defense balance by coordinating hormone biosynthesis and signaling pathways. Experimental studies have demonstrated that disruption of BR or GA signaling reduces disease resistance and plant growth, indicating that both hormones are required for optimal stress adaptation [83,96,97,98]. At the molecular level, BR–auxin coordination involves regulation of auxin transport components, auxin-responsive transcription factors, and shared downstream targets controlling cell expansion. Similarly, BR-GA interactions influence growth through coordinated regulation of biosynthetic genes and transcriptional regulators involved in cell elongation and developmental transitions.

### 7.5. Network-Level Integration of Hormonal Signaling

Plant hormone signaling operates as an integrated network rather than independent pathways. BRs represent one important component within this network by interacting with multiple hormones simultaneously to regulate gene expression, metabolism, and stress responses [83]. Rather than functioning as a hierarchical controller of other hormones, BR signaling participates in reciprocal feedback networks where hormone pathways influence each other’s biosynthesis, signaling activity, and downstream transcriptional responses. Importantly, BR–hormone interactions can be either positive or negative and are highly dependent on stress type, developmental stage, tissue specificity, and hormone concentration. Positive interactions can enhance stress tolerance through coordinated activation of antioxidant defense, osmotic adjustment, defense-related gene expression, and cross-regulation of hormone biosynthesis and signaling. In contrast, negative interactions can restrain growth, modify developmental processes, or attenuate excessive defense responses, thereby reducing the metabolic costs associated with stress adaptation. For example, although BR signaling generally promotes growth, its interaction with ABA, JA, SA, or ethylene may redirect resources from growth toward stress protection under adverse conditions. Thus, the same hormonal interaction may produce different outcomes depending on the physiological context. The final physiological outcome depends on the relative strength, timing, and spatial activation of each hormonal pathway [83,99]. This integrated network enables plants to balance growth, defense, and stress adaptation under changing environmental conditions.

## 8. BRs in Crop Improvement and Agriculture Application

BRs have emerged as promising tools for improving crop productivity and resilience under adverse environmental conditions. However, the current application of BR-based technologies remains largely at the experimental validation stage. Most reported beneficial effects of BR treatments have been obtained under controlled greenhouse or laboratory conditions, whereas evidence from multi-location and multi-season field trials remains comparatively limited. Therefore, although BRs represent promising regulators of crop stress tolerance, their commercial implementation requires further evaluation considering crop genotype, BR formulation, application concentration, developmental stage, environmental conditions, and economic feasibility. These phytohormones regulate plant growth, development, and stress responses through coordinated modulation of physiological, biochemical, and molecular processes. In recent years, significant attention has been directed toward the agricultural application of BRs to enhance crop performance, particularly under drought, salinity, temperature extremes, and heavy metal stress conditions [12,64]. The integration of BR-based strategies into crop management practices has demonstrated substantial potential for improving stress tolerance, yield stability, and resource-use efficiency. However, practical implementation of these strategies requires careful consideration of dosage, timing, crop species, and environmental factors to achieve consistent field performance.

### 8.1. Exogenous Application of BRs for Stress Tolerance

Exogenous application of BRs has been widely investigated as a promising approach to enhance plant tolerance to environmental stresses, particularly under controlled experimental conditions. Foliar spraying, seed priming, and soil application of BRs have been shown to improve plant growth, photosynthetic efficiency, and stress resistance in various crop species [64]. These treatments stimulate antioxidant enzyme activity [64]. Improve membrane stability and promote osmolyte accumulation, thereby protecting plants from stress-induced damage [64]. Under drought and salinity stress conditions, BR application enhances water-use efficiency and maintains ion balance by regulating stomatal conductance and ion transport mechanisms. Additionally, BR-treated plants often exhibit improved root growth and nutrient uptake, which contribute to enhanced stress tolerance and crop productivity [10,49,51]. Experimental studies have demonstrated that exogenous BR application can improve plant survival, growth, and physiological performance under various stress conditions. However, translation of these beneficial effects into consistent agricultural outcomes requires further field validation across diverse environments and crop production systems.

### 8.2. Genetic Manipulation of BR Biosynthesis and Signaling Genes

Genetic engineering of BR biosynthesis and signaling pathways represents another promising approach for improving crop performance. Manipulation of key genes involved in BR biosynthesis, such as *DWF4*, *CPD*, and *BR6ox*, can alter endogenous BR levels and influence plant growth, development, and stress tolerance; however, the optimal level of BR accumulation is highly dependent on species, tissue type, developmental stage, and environmental conditions. Similarly, modification of signaling components such as BRI1, BIN2, and BZR1 can influence plant architecture, biomass accumulation, and stress resistance, although excessive activation or suppression of these pathways may cause trade-offs between growth, reproduction, and stress adaptation. [20,41,100]. Transgenic plants with enhanced BR signaling often exhibit improved tolerance to drought, salinity, and temperature stress due to increased antioxidant capacity and improved cellular homeostasis. However, excessive BR accumulation may lead to undesirable phenotypes such as elongated stems and reduced reproductive performance, highlighting the need for balanced regulation of BR pathways in crop improvement programs. Recent advances in genome editing technologies, including CRISPR/Cas systems, have enabled precise modification of BR-related genes to optimize plant growth and stress responses [20]. However, improving crop performance through BR manipulation requires fine-tuning of BR pathways rather than simply increasing BR biosynthesis or signaling activity. Emerging strategies, including promoter editing, partial-loss-of-function alleles, tissue-specific regulation, stress-inducible expression, and developmental-stage-specific modulation, may provide more precise control of BR responses while minimizing undesirable effects on plant architecture and reproduction. In addition, editing specific BR-related paralogs or modifying receptor sensitivity may allow targeted adjustment of BR signaling in particular tissues or environmental conditions. These precision approaches could facilitate the development of crops with improved stress resilience while maintaining optimal growth and yield performance.

### 8.3. BRs in Enhancing Yield Stability Under Stress

Yield stability is a critical objective in modern agriculture, particularly under changing climate conditions. BRs contribute to yield stability by improving plant growth, reproductive development, and stress tolerance. Studies have shown that BR-treated crops maintain higher grain yield and biomass production under stress conditions compared with untreated plants [64]. BR-mediated yield improvement is associated with enhanced photosynthesis, improved nutrient utilization, and increased resistance to environmental stress. In addition, BR signaling regulates flowering time, seed development, and fruit set, which are essential determinants of crop yield under stress conditions [12,101]. Field studies in cereals, legumes, and horticultural crops have provided encouraging evidence that BR application may improve yield stability under drought and salinity stress conditions. However, differences among crop species, BR formulations, application methods, environmental conditions, and experimental duration highlight the need for broader multi-location and multi-season evaluations before large-scale agricultural adoption [64].

### 8.4. Challenges and Limitations in Field Applications

The major BR-based strategies for improving crop stress tolerance, along with their target mechanisms, advantages, and limitations, are summarized in Table 2. Despite the promising potential of BRs in crop improvement, several challenges limit their widespread adoption in agriculture. One major limitation is the variability in plant response to BR application, which depends on factors such as crop species, developmental stage, environmental conditions, and application method [49,102]. Another challenge is the relatively high cost of synthetic BR compounds, which may restrict large-scale agricultural use. In addition, excessive BR application can lead to abnormal plant growth and reduced stress tolerance, emphasizing the importance of optimizing dosage and timing for effective field application [49]. Environmental factors such as soil type, temperature, and water availability can also influence the effectiveness of BR treatment, making it necessary to develop crop-specific management strategies. Addressing these challenges will be essential for translating laboratory findings into practical agricultural applications [24].

Although exogenous BR application has shown considerable promise for improving crop productivity and stress tolerance, its large-scale agricultural adoption depends on economic feasibility (Table 3). Foliar spraying is currently the most practical and cost-effective application method because it requires relatively small quantities of BRs and can be readily integrated into existing crop management practices. Seed priming offers an additional economical approach, as a single pre-sowing treatment can enhance seedling establishment and stress resilience while minimizing repeated applications. In contrast, repeated foliar applications throughout the growing season increase labor and production costs, potentially limiting their adoption in large-scale farming systems.

Another important limitation is the environmental stability of exogenously applied BRs. These compounds are susceptible to degradation through sunlight, microbial activity, and wash-off by rainfall or irrigation, reducing their persistence and biological efficacy under field conditions. Consequently, maintaining effective BR concentrations often requires multiple applications, increasing production costs and reducing cost-effectiveness. Future research should therefore focus on developing more stable and efficient BR delivery systems, including nano-formulations, controlled-release carriers, and encapsulation technologies that protect BRs from environmental degradation and prolong their activity. In parallel, genetic approaches aimed at enhancing endogenous BR biosynthesis or signaling may provide a more sustainable long-term alternative by reducing dependence on repeated external applications.

## 9. Emerging Trends and Future Perspectives

Throughout this review, several recurring limitations become evident. While substantial progress has been made in elucidating BR biosynthesis, signaling, and stress responses, major challenges remain in defining missing signaling components, validating candidate genes identified through omics approaches, understanding hormone crosstalk under combined stresses, translating greenhouse findings into field conditions, and minimizing pleiotropic effects associated with manipulating BR signaling. The following sections outline future research priorities that directly address these unresolved questions. Research on BRs has advanced rapidly over the past decade, revealing their central role in regulating plant growth, development, and stress responses. Recent technological innovations, particularly in systems biology, genomics, and editing, have significantly expanded our understanding of BR signaling networks and their applications in crop improvement. These advances are expected to facilitate the development of stress-resilient crops capable of maintaining productivity under changing environmental conditions. Future research on BR biology will increasingly focus on integrating multi-omics datasets, understanding hormone interactions under complex environmental conditions, and translating molecular knowledge into practical agricultural applications. Addressing these emerging challenges will be essential for improving crop sustainability and food security in the face of global climate change.

### 9.1. System Biology and Omics Approaches

System biology and omics technologies, including genomics, transcriptomics, proteomics, metabolomics, and phenomics, have transformed the study of plant hormone signaling. These approaches enable comprehensive analysis of BR-mediated regulatory networks and identification of key genes and pathways involved in stress tolerance and plant development. Transcriptomics and proteomic studies have revealed that BR signaling regulates thousands of genes associated with growth, metabolism, and stress adaptation. However, despite these advances, the upstream regulatory hierarchy linking BR receptors to BIN2/BZR1 signaling, downstream transcription factors, and metabolic reprogramming remains incompletely understood. In particular, the temporal coordination of BR signaling with ABA, ethylene, JA, and SA pathways during stress progression is still poorly resolved. Future integration of spatial transcriptomics, single-cell RNA sequencing, quantitative phosphoproteomics, and metabolomics will help identify these missing signaling nodes and clarify how BR-dependent regulatory networks are dynamically reorganized during stress adaptation. Metabolomic analysis further demonstrated that BR treatment influences the accumulation of metabolites involved in antioxidant defense, osmotic adjustment, and energy metabolism [78]. Integration of multi-omics data provides valuable insights into the complex interactions between BR signaling pathways and other hormonal networks, facilitating the identification of novel targets for crop improvement and stress management.

### 9.2. BR Signaling Under Combined Biotic and Abiotic Stresses

Plants in natural environments are often exposed to multiple stresses simultaneously, such as drought combined with pathogen infection or salinity combined with temperature stress. However, most BR studies summarized in this review have investigated individual stress factors under controlled experimental conditions. In contrast, crops grown in agricultural fields frequently encounter simultaneous drought, heat, salinity, and pathogen attack, where stress interactions can substantially alter physiological and molecular responses. Whether BR signaling coordinates these combined stress responses through common regulatory modules or stress-specific signaling pathways remains largely unknown. Recent studies indicate that BR signaling plays a crucial role in coordinating plant responses to combined stress conditions by regulating gene expression, antioxidant activity, and hormonal interactions [10]. Under combined stress conditions, BR signaling modulates defense responses to maintain cellular homeostasis and minimize damage. For example, BR-treated plants have been shown to exhibit enhanced tolerance to simultaneous drought and pathogen stress through improved antioxidant defense and activation of stress-responsive genes [106]. Future studies should prioritize factorial experiments that combine multiple abiotic and biotic stresses with time-resolved transcriptomic, proteomic, and physiological analyses to identify BR-dependent regulatory networks unique to combined stress conditions. Understanding BR-mediated responses to combined stress is essential for developing crops capable of thriving under complex environmental conditions associated with climate change.

### 9.3. Natural Variation and BR-Related Quantitative Trait Loci (QTLs)

Natural genetic variation in BR-related genes plays an important role in determining plant growth, stress tolerance, and yield performance. Identification of QTLs associated with BR biosynthesis and signaling has provided valuable insights into the genetic basis of stress tolerance in crops [107]. Recent genomic studies have identified BR-related QTLs linked to traits such as drought tolerance, plant height, grain yield, and disease resistance in major crops including rice, wheat, and maize. However, although several BR-related QTLs have been reported across these crop species, relatively few have been functionally validated through gene editing, mutant analysis, or complementation experiments. Consequently, the causal genes underlying many reported loci remain uncertain, limiting their effective utilization in molecular breeding programs. These discoveries highlight the potential of using natural genetic variation to develop improved crop varieties through marker-assisted selection and breeding programs [108]. Future research should integrate genome-wide association studies (GWAS), high-density QTL mapping, haplotype analysis, and CRISPR/Cas-mediated functional validation to accelerate the identification of causal BR regulatory genes suitable for precision breeding. Advances in genome sequencing and bioinformatics tools have further facilitated the identification of BR-related genetic markers, enabling more efficient breeding strategies for stress-resilient crops.

### 9.4. Translational Potential for Climate Resilient Crops

The increasing frequency of extreme weather events and environmental stresses has intensified the need for climate-resilient crop varieties. BRs offer significant potential for improving crop performance under challenging environmental conditions due to their ability to regulate growth, stress tolerance, and resource-use efficiency [7,109]. Application of BR-based technologies, including hormone treatment, genetic engineering, and genome editing, has demonstrated promising results in enhancing crop productivity and resilience. However, although exogenous BR application has consistently improved stress tolerance under controlled greenhouse conditions, its performance under field conditions remains inconsistent. Treatment efficacy varies among crop species, developmental stages, stress intensity, environmental conditions, formulation stability, and application timing, limiting the widespread adoption of BR-based technologies in agricultural practice. For example, manipulation of BR biosynthesis and signaling pathways has been shown to improve drought tolerance, salinity resistance, and nutrient efficiency in several crop species. Future research should focus on optimizing BR formulations, developing controlled-release delivery systems, refining application schedules, and establishing crop-specific dosage recommendations through multi-location field trials. Integration of BR research into crop breeding programs will be critical for developing sustainable agricultural systems capable of meeting future food demands.

### 9.5. Knowledge Gaps and Research Priorities

Despite substantial progress in elucidating BR biosynthesis, signaling, and stress adaptation, several important knowledge gaps continue to limit the effective application of BR-based technologies in crop improvement. Although the core BR signaling pathway has been well characterized, the regulatory mechanisms linking BR perception by the BRI1/BAK1 receptor complex to stress-specific transcriptional networks remain incompletely understood. Likewise, many BR-responsive candidate genes identified through transcriptomic and other omics studies have not been functionally validated, leaving the causal roles of numerous regulatory components unresolved. The molecular basis of BR crosstalk with ABA, JA, SA, and ethylene under combined biotic and abiotic stresses also remains poorly understood. Furthermore, most mechanistic evidence has been generated in model species such as *Arabidopsis thaliana* and rice, whereas many economically important cereal, legume, and horticultural crops remain underexplored. From an applied perspective, inconsistent field performance, limited understanding of the long-term ecological and economic impacts of repeated BR application, and the pleiotropic effects associated with manipulating BR signaling continue to hinder agricultural deployment. Future research should therefore integrate advanced molecular biology, multi-omics, functional genomics, genome editing, quantitative genetics, and multi-location field validation to develop precise, efficient, and sustainable BR-based strategies for climate-resilient crop improvement.

## 10. Conclusions

BRs are essential plant hormones that play a central role in regulating growth, development, and stress responses. Extensive research has demonstrated that BR signaling influences a wide range of physiological and molecular processes, including cell elongation, antioxidant defense, hormone interactions, and gene expression. These mechanisms enable plants to adapt to diverse environmental stresses and maintain productivity under adverse conditions. The integrative and context-dependent role of BRs in coordinating plant responses to abiotic and biotic stresses highlights their importance in plant stress physiology and crop improvement. Through dynamic interactions with other phytohormones and signaling pathways, BRs contribute to the regulation of complex networks that coordinate plant growth, development, and defense under changing environmental conditions. Advances in biotechnology, genomics, and agricultural management practices have expanded the potential application of BRs in sustainable agriculture. Nevertheless, successful application of BR-based technologies requires careful optimization because BR responses vary with crop species, developmental stage, environmental conditions, application strategy, and hormone dosage. Moreover, excessive BR signaling or application may result in undesirable effects on plant architecture, reproduction, or yield, while evidence from large-scale multi-location field studies remains limited. Continued research integrating molecular biology, genetics, physiology, and field-based validation will be essential to optimize BR-mediated strategies, minimize potential trade-offs, and develop climate-resilient crops capable of sustaining productivity under changing environmental conditions.

## Figures and Tables

**Table 1:** Major Brassinosteroid Biosynthetic and Metabolic Genes and their Functions.

Gene	Enzyme Type	Function	Role in Pathway	References
DWF4	Cytochrome P450 (CYP90B1)	C-22 hydroxylation	Rate-limiting biosynthesis step	[29]
CPD	Cytochrome P450 (CYP90A1)	C-23 hydroxylation	Intermediate formation	[29]
DET2	Steroid reductase	Reduction of sterol intermediates	Early biosynthesis step	[30]
ROT3 (CYP90C1)	Cytochrome P450	C-23 hydroxylation	Intermediate conversion	[31]
CYP90D1	Cytochrome P450	C-23 hydroxylation	Late biosynthesis step	[31]
BR6ox1/2	Cytochrome P450 (CYP85A1/CYP85A2)	C-6 oxidation of 6-deoxo BR intermediates/conversion of castasterone to brassinolide	Late and final BR biosynthesis step	[32,33]
BAS1	Cytochrome P450 (YP734A1)	C-26 Hydroxylation of brassinolide	Catabolism	[26,34]

**Table 2:** Conserved and stress-specific mechanism of brassinosteroid mediated abiotic stress tolerance.

Abiotic Stress	Conserved BR-Mediated Responses	Stress-Specific BR-Mediated Mechanisms	Representative Molecular Components
Drought	ROS homeostasis, membrane protection, osmotic adjustment, transcriptional reprogramming	Stomatal closure, improved water-use efficiency, ABA crosstalk	BZR1/BES1, ABA signaling components, DWF4, CPD
Salinity	ROS detoxification, membrane stability, osmolyte accumulation	Na^+^/K^+^ homeostasis, regulation of ion transporters	SOS pathway, NHX transporters, HKT transporters
Low temperature	ROS scavenging, membrane stabilization, metabolic adjustment	Cold acclimation, CBF-dependent and CBF-independent pathways, maintenance of membrane fluidity	CBF/COR genes, BZR1
High temperature	Antioxidant defense, maintenance of cellular homeostasis	Heat-shock protein induction, protein stabilization, thermotolerance	HSPs, HSFs, BZR1/BES1
Heavy metal stress	ROS detoxification, antioxidant defense	Metal chelation, vacuolar sequestration, detoxification	Phytochelatins, metallothioneins, ABC transporters
Oxidative stress	Activation of antioxidant enzymes, redox homeostasis	Direct regulation of ROS-scavenging and antioxidant metabolism	SOD, CAT, POD, APX, GR

**Table 3:** BR-Based Strategies for Crop Stress Tolerance.

Strategy	Target Mechanism	Stress Type	Crop Response	Advantages	Limitations	References
Foliar application of BRs	Activation of antioxidant enzymes and osmolyte synthesis	Drought, salinity, heavy metal	Improved stress tolerance and growth	Simple and rapid application	Variable field response	[49]
Seed priming with BRs	Enhanced germination and early growth	Drought, temperature stress	Increased seedling vigor	Improved establishment	Dose-dependent effects	[103]
Genetic engineering of BRs biosynthesis genes	Increased endogenous BR production	Multiple stresses	Enhanced growth and stress resistance	Long-term improvement	Regulatory and biosafety concerns	[104,105]
Integrated hormone management	Coordination of multiple hormone pathways	Combined stresses	Enhanced stress adaptation	Sustainable crop production	Requires optimized protocols	[49]

## Data Availability

All data supporting the findings of this study are included within the article.
